# A hybrid approach to predicting and classifying dental impaction: integrating regularized regression and XG boost methods

**DOI:** 10.3389/froh.2025.1524206

**Published:** 2025-04-28

**Authors:** Asok Mathew, Pradeep K. Yadalam, Ahmed Radeideh, Shrouk Hady, Rona Swed, Reyyan Cheema, Majd Mousa AL-Mohammad, Mohammed Alsaegh, SR Shetty

**Affiliations:** ^1^ Department of Clinical Sciences, College of Dentistry, Centre for Medical and Bio-Allied Health Sciences Research, Ajman University, Ajman, United Arab Emirates; ^2^ Department of Periodontics, Saveetha Dental College, SIMATS, Saveetha University, Chennai, Tamil Nadu, India; ^3^ Department of University requirements, University of Fujairah, Fujairah, United Arab Emirates; ^4^ Department of oral and craniofacial Health Sciences, College of Dental Medicine, University of Sharjah, Sharjah, United Arab Emirates

**Keywords:** dental impaction, artificial intelligence, oral surgery, machine learning, ensemble learning

## Abstract

**Introduction:**

Dental impaction is a significant clinical challenge that requires advanced predictive modeling and healthcare analytics approaches. Impaction, a tooth alignment issue, is diagnosed using radiographic measurements like panoramic radiographs and CBCT. Artificial Intelligence (AI) is improving the accuracy of predicting dental impaction. Advanced predictive models like logistic Regression and XGBoost analyze critical variables, identify patterns, and perform predictive analysis. These models can identify potential impactions, assess impaction type, and develop treatment plans. Integrating AI into radiographic assessments is expected to enhance further the precision and risk-minimizing capabilities of surgical planning in dentistry. This study presents a hybrid approach combining regularized regression and ensemble methods to enhance the classification and prediction of dental impaction outcomes. By leveraging machine learning and statistical learning techniques, we aim to develop a robust clinical decision support system for dental practitioners.

**Methods:**

This research aims to predict the eruption of 3rd molars in the mandible by analyzing three parameters: the distance from the lower 2nd molar to the anterior border, the mesiodistal width of the third molar, and the distance from the apex of the root to the inferior border of the mandible. The study is quantitative, observational, and cross-sectional retrospective. The distance from the lower 2nd molar to the anterior border determines the importance of space available for eruption. The distance from the root apex to the lower border addresses natural eruptive forces and resistance during the eruption. The study aims to find a correlation between eruption and distance from the root apex to the lower border of the mandible. Our feature selection process utilizes ensemble learning algorithms integrated with regularized regression techniques to analyze various parameters. This data analysis framework combines multiple predictive modeling approaches to achieve optimal results.

**Results:**

The horizontal type of impaction has the lowest S/W ratio (0.9267), indicating the least available distal to 2nd molar space. This suggests a low potential for future eruptions. The regression equation calculates the S/W ratio using impacted molar width and distal space. A ratio greater than 1.1 indicates a good probability of lower 3rd molar eruption, while a below 0.8 indicates no eruption. The algorithm development process demonstrated the effectiveness of our hybrid approach in dental health analytics. The study improved impaction prediction accuracy to a rate of 78%, with horizontal class predictions achieving a precision of 0.72 and an error rate of 28.1%. Additionally, the regularized logistic regression model attained 75% accuracy for classification and prediction.

**Conclusion:**

The study aims to improve dental research by predicting the eruption behavior of lower molars, enabling dental practitioners to make more concise treatment plans. The study identifies the most significant parameters for establishing the space/width ratio: Distance from the second molar to the anterior ramus border and the third molar's mesiodistal width. Enhancing data quality, refining feature selection, and using advanced modeling techniques are crucial for improving predictive capabilities. The findings can help practitioners optimize treatments and reduce potential complications.

## Introduction

Impaction is the improper alignment of a tooth within the alveolar bone, preventing it from erupting into its normal position. It is more prevalent with third molars and can occur with other teeth. Radiographic measurements, such as panoramic radiographs and CBCT, are crucial in diagnosing and assessing impaction. These measurements provide information about the impacted tooth's size, position, angulation, and orientation, aiding in treatment planning and decision-making (1). The angulation of the impacted tooth relative to the dental arch determines the difficulty of extraction and whether orthodontic treatment may be necessary. The depth of impaction determines whether the tooth is fully impacted or partially exposed (2).

A prediction on the future eruption/impaction can be made based on the selected parameters, such as the width of the third molar, the space available distal to the second molar, and the distance from the apex to the lower border. If the width of the third molar is larger than the available space distal to the second molar, it suggests a high probability of impaction(i.e., the S/W ratio is less than 1). The third molar may not erupt due to insufficient space in the dental arch and a smaller distance from the apex to the lower border, indicating a vertical impaction (3). This could lead to extraction or additional treatment to prevent complications like inflammation, infection, or damage to adjacent teeth. However, a comprehensive clinical examination and radiographic assessment by a dentist or oral surgeon is necessary for accurate diagnosis and prediction of impaction, as these parameters alone are insufficient. One previous study performed predictive analysis on 200 subjects using digital panoramic radiographs. It revealed significant differences in lower eruption space measurements, α-angle, and β-angle, with males having more values than females. These measurements provide accurate information for predicting lower third molar eruption or early impaction (4).

Artificial Intelligence (AI) revolutionizes medicine and dentistry by aiding disease diagnosis, prognosis, and prediction. AI has been shown to detect diseases like coronary artery calcification, cerebral microhemorrhages, diabetic retinopathy, and breast or skin cancer. Recent advancements in machine learning and deep learning techniques have significantly improved the accuracy of predicting dental impaction. Accurate prediction aids dental professionals in planning preventive measures, reducing healthcare costs, and enhancing patient outcomes (5). Traditional dental impaction diagnosis methods often rely on clinical evaluations and imaging techniques. Advanced predictive models, like logistic Regression and XGBoost, can enhance the diagnostic process by analyzing critical variables, identifying patterns, and providing predictive results based on historical data (6).

Venta et al. (1997) (7) devised a method to predict impaction. They measured the distance from the distal of the 2nd molar to the anterior ramus. The results illustrated that if the distance between the distal surface of the lower 2nd molar C anterior ramus is less than or equal to 9.5 mm, the probability of impaction is found to be 100%, and if it is less than 14.5 mm, impaction probability is 76%. The distance between the distal of the lower 2nd Molar C Anterior Ramus is more than 14.5 mm, and the probability of eruption is 72%. The distance between the lower 2nd Molar C Anterior Ramus distal is greater than 16.5 mm, and eruption probability is 100%.

A previous study by Mahmut Emin Celik (8) on 440 panoramic radiographs from 300 patients used Faster RCNN with ResNet50, AlexNet, VGG16, and YOLOv3. YOLOv3 showed the highest detection efficacy, recall, and precision for impacted mandibular third molar tooth detection, demonstrating the reliability and robustness of diagnostic tools. Another study analyzed 1864 mandibular third molar images, analyzing impaction patterns using Pell and Gregory and Winter classifications. The ML classification model for mandibular third molar impaction status showed good performance with accuracy, F1-score, and AUC values ranging from 0.7959–0.9549 when data augmentation techniques were applied. However, these studies were not based on radiographic measurements.

Predictive models can identify potential impactions, assess impaction type, and develop treatment plans (9, 10). Standardized classification systems facilitate clear communication among dental professionals, improving patient education. Predictive analyses can lead to more efficient treatment pathways and reduce treatment time. Integrating machine learning and AI into radiographic assessments will further enhance this approach's significance in dentistry (11, 12). Due to their advantages, logistic Regression and XGBoost are effective methods for predicting dental impaction outcomes. Logistic Regression provides interpretability, produces probabilities, and is efficient with linearly separable data. It is less susceptible to overfitting and is a baseline for more complex models. XGBoost, on the other hand, is known for its high predictive accuracy and speed, particularly with large datasets. It can handle non-linearity, feature importance, robustness to overfitting, and scalability (13, 14). So, we used these algorithms to better interpret predictive results for radiographic measurements.

This study utilizes regularized logistic Regression and XGBoost (15, 16) to enhance dental impaction prediction accuracy, capturing linear relationships and addressing complex non-linear interactions. Machine learning is crucial for predicting dental impaction due to its efficient processing of large datasets and ability to identify hidden patterns. It can learn intricate relationships among features like age, gender, and medical history, paving the way for more accurate, proactive, and personalized patient care. The study aims to predict tooth impact from OPG radiographs using linear parameters, identify margins for eruption, determine regression equation accuracy, and compare logistics regression and extreme Gradient boosting models.

## Methodology

The study was approved by the ethical committee of Ajman University (D-H-S-2023-NOV-03-5). This research was carried out to predict impaction or eruption of 3rd molars in the mandible. This prediction will be built on the analyses of three parameters:

• The distance from the lower 2nd molar to the most anterior border of the mandible,
• The mesiodistal width of the third molar,
• And the distance from the root's apex to the mandible's inferior border.

**Figure 1:**
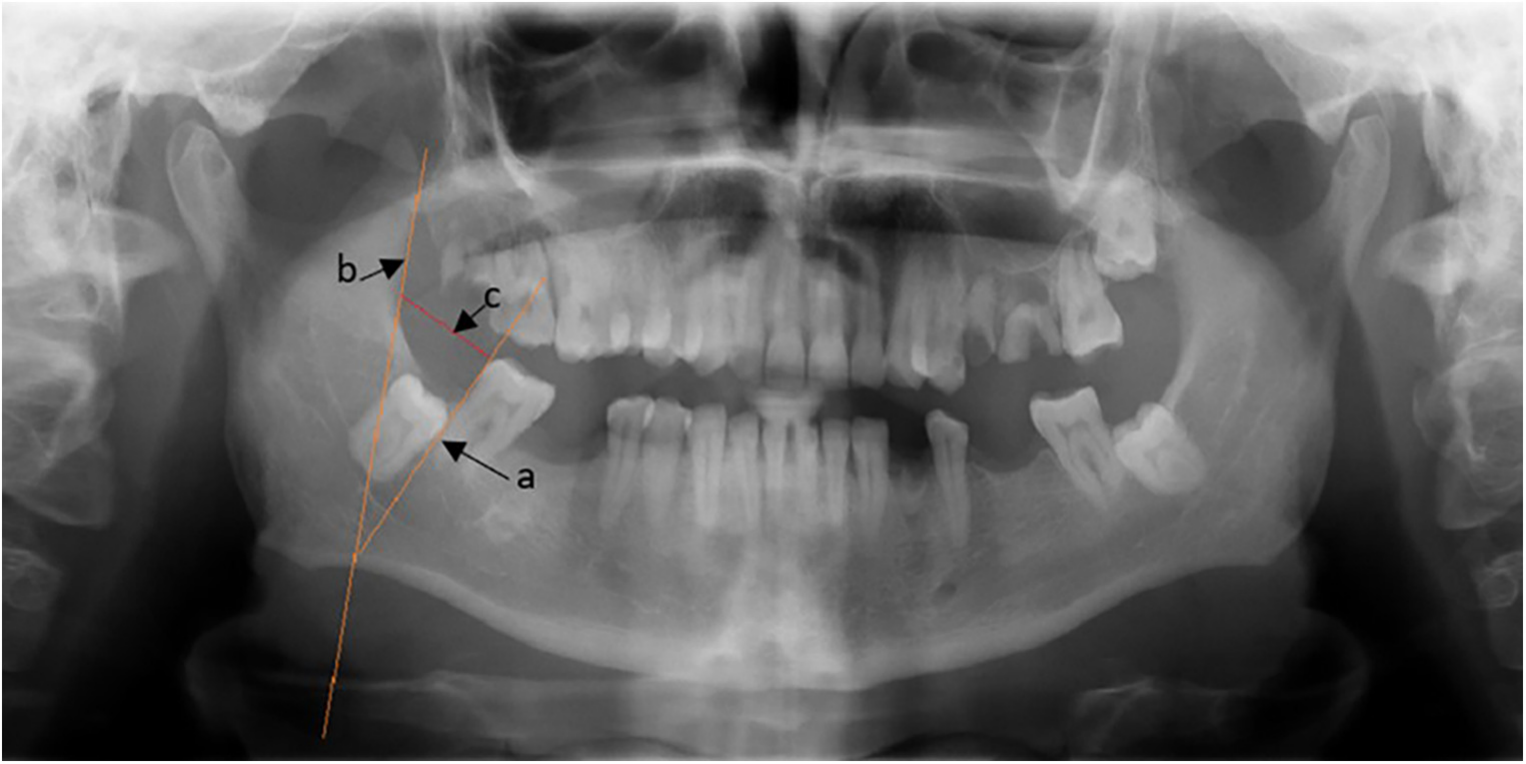
Distance from the ramus border to the second molar's distal aspect.

**Figure 2:**
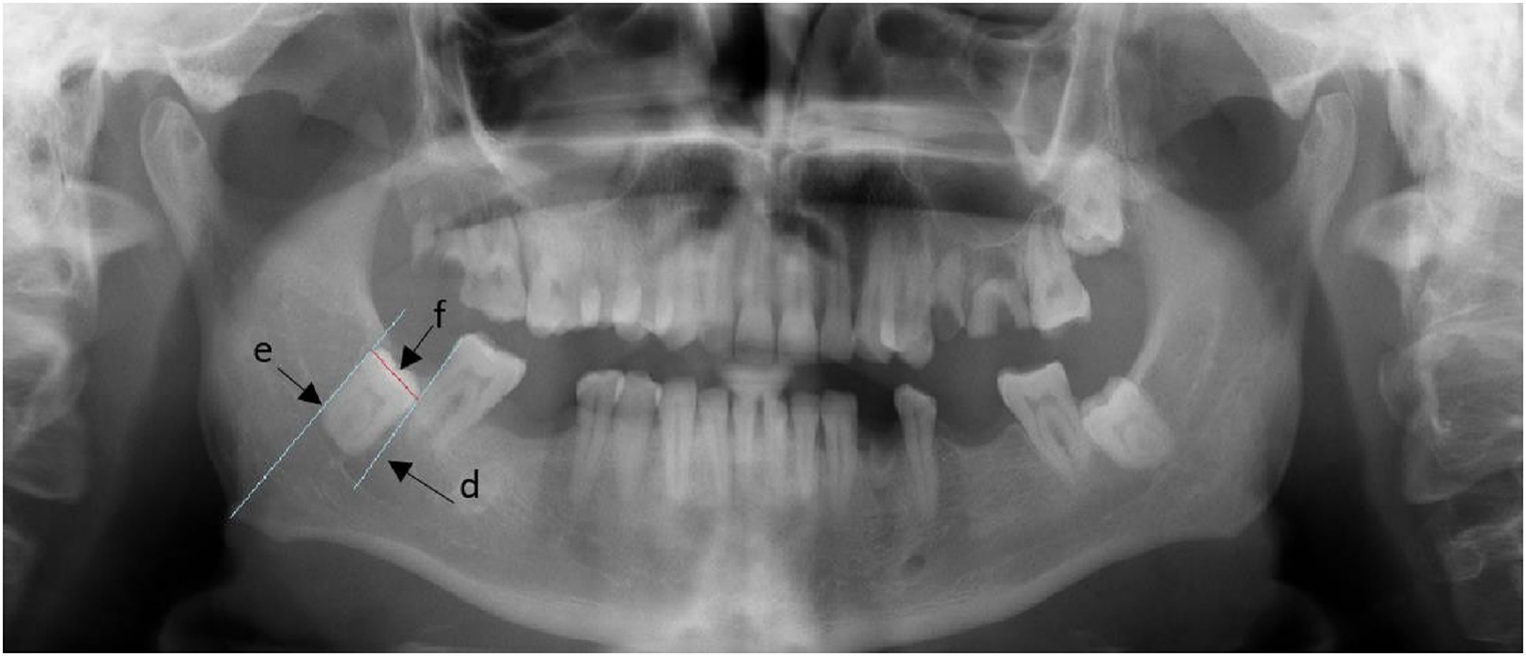
Shows the measurement of the mesiodistal width of the third molar.

**Figure 3:**
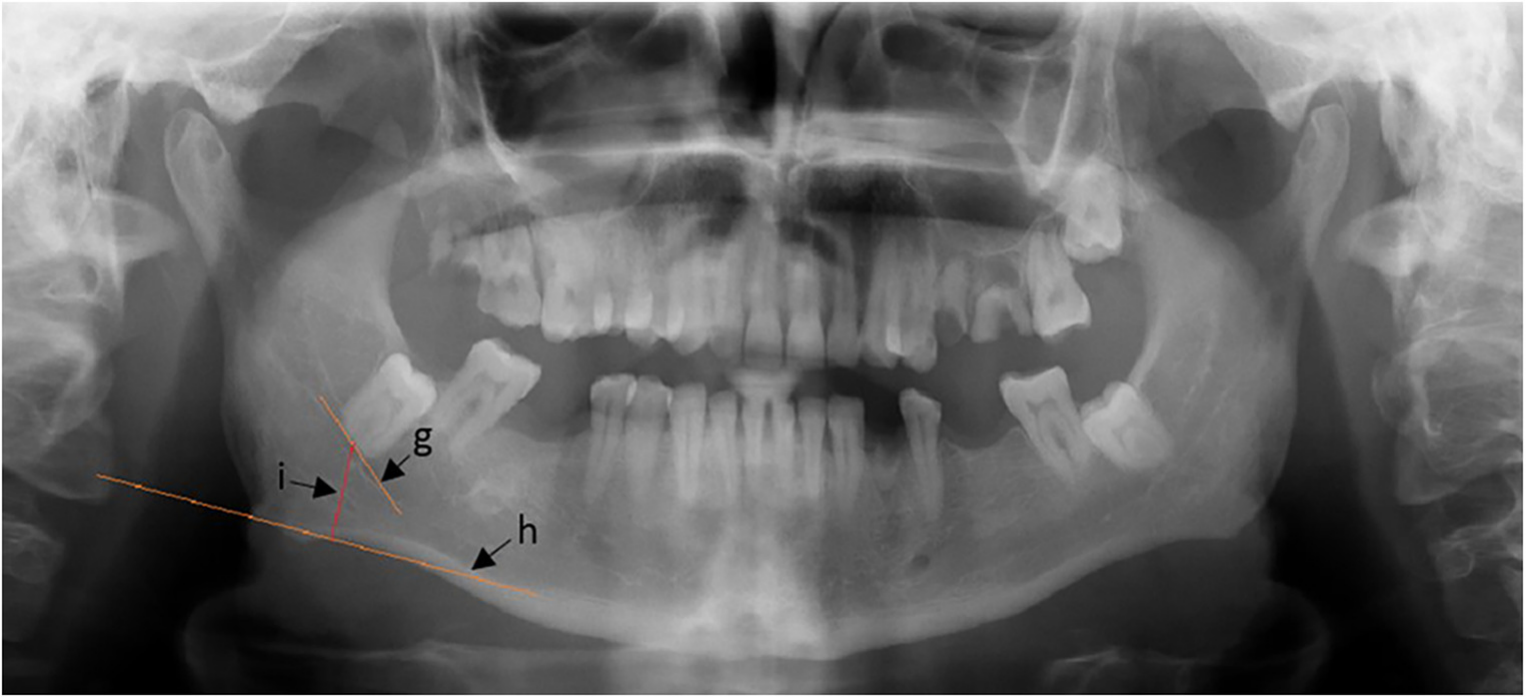
Distance from the root's apex to the mandible's inferior border.

This study is a quantitative, observational, cross-sectional, retrospective analysis with a sample size of 303 OPGs. The first parameter is the distance from the lower 2nd molar to the anterior border of the mandible. This parameter is observed and studied to see the importance of the space available. When the dimension from the second molar's distal to the mandible's anterior border is calculated and determined, it will be divided by the mesiodistal width of the unerupted third molar, and a ratio will be obtained. As was also previously mentioned, if the ratio is greater than 1.1, the chances of eruption are good, and if the ratio is less than 0.8, no chance of eruption exists (Figures 1–3). The third parameter is the distance from the apex of the root of the lower third molar to the lower border of the mandible, and this parameter will be used to address the natural eruptive forces acting on the tooth vs. the resistance faced during an eruption as the roots get formed. While we haven't found studies that mention a correlation between eruption and distance from the root apex to the lower border of the mandible, we will be trying to find a link to whether this correlation is relevant in this study.

The exclusion criteria originally were Images with any artifacts, Images with any bony pathology obscuring the region of interest, and images that are unclear/without any proper details of the set parameters.

After the data is sorted, the variables will be measured using Scanora 5.2.6 and measured in millimeters using the viewing software. The x-rays were taken under the following conditions: 70 kV and 10 mA. For the first parameter, as is depicted in Figure 1, we constructed a line adjacent to the distal surface of the second molar surface (line a) and a line adjacent to the anterior border of the ramus (line b). We construct a line connecting them at the level of the occlusal surface of the second molar (line c). These measurements are obtained by an independent dental clinician specializing in evaluating impaction through Orthopantomography (OPG).

For the second parameter, as shown in Figure 2, we construct a line adjacent to the mesial surface of the third molar (line d), a line adjacent to the distal surface of the third molar (line e), and a line connecting them at the occlusal surface, (line f) (line f) will be measured. The measurement will be used for the second parameter.

Lastly, for the third parameter, as shown in Figure 3, a line will be constructed perpendicular to line h, which will be constructed on the inferior border of the mandible (line h). Then, a line will be constructed between them, (line i), and the length of (line i) will be the measurement of the third parameter.

Two of the four investigators measured these variables for each of the OPGs. Measurements will be done individually to avoid influencing the other observer's reading. The results will be analyzed and then compared to the figures mentioned previously. A regression equation will be plotted to assess the strength of the association and check its statistical significance.

### Machine learning

#### Data preparation

**Figure 4:**
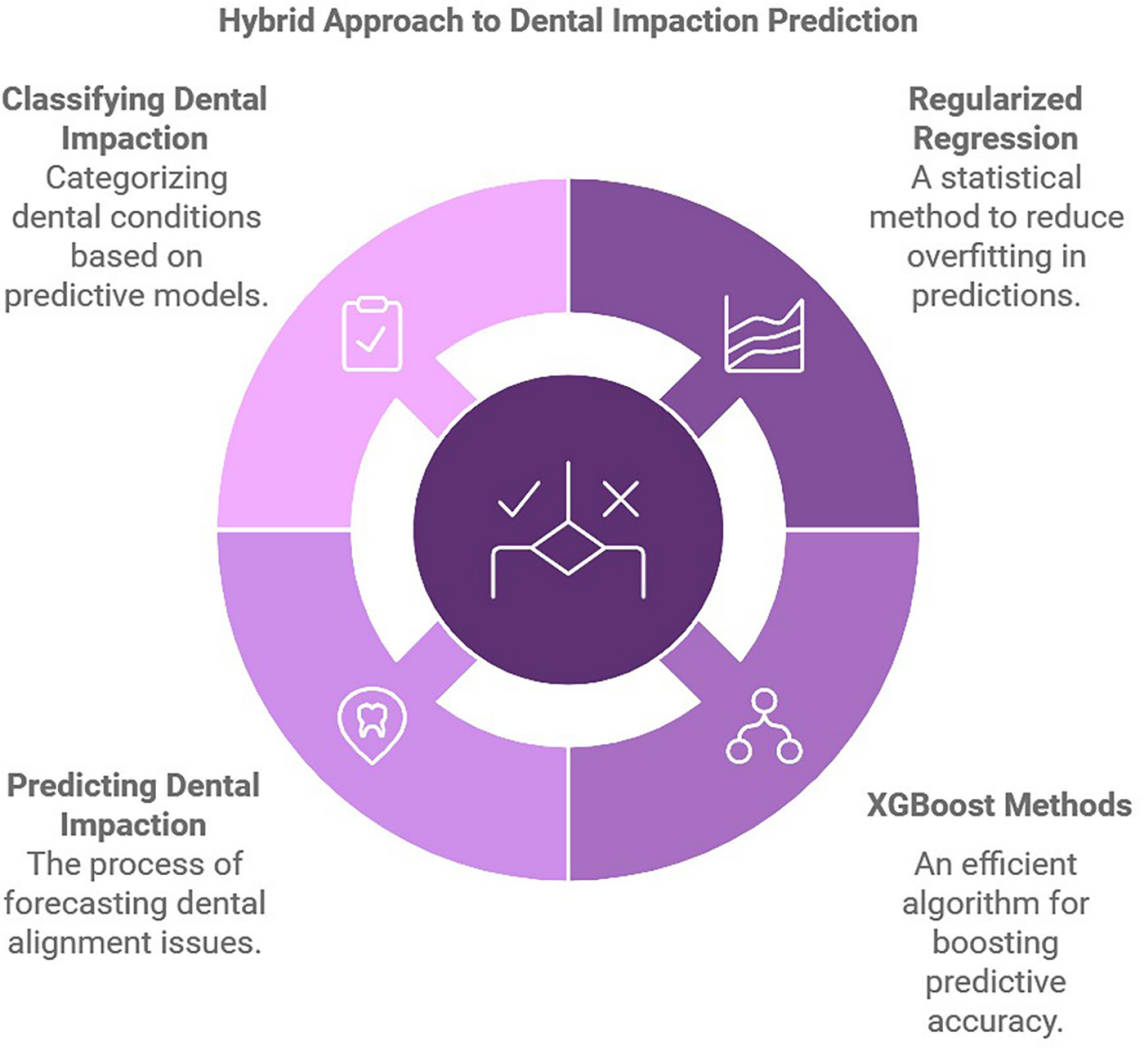
Workflow of the AI model.

Data obtained from investigators were subjected to preprocessing steps like removing missing values normalization and were split into 80 percent train and 20 percent test data. Using the datarobot tool, data were subjected to logistics regression and extreme Gradient boosting (Figure 4).

#### Regularised logistics regression architecture

Regularized logistic Regression (17) is a machine learning algorithm used for binary classification tasks, incorporating a regularization term into the cost function to prevent overfitting and improve model generalization capabilities. Regularized Logistic Regression is an extension of logistic Regression that introduces a penalty term to the loss function to prevent overfitting and enhance model generalization. While standard logistic Regression focuses solely on minimizing the logistic loss, regularization can help constrain the model's complexity by discouraging overly large coefficients (Table 1). Logistic Regression is a binary.

**Table 1:** Shows hyperparameters used in this model.

Model	Hyperparameter	Value/Description
Regularized Logistic Regression	Regularization Strength (*λ*)	Adjusted via cross-validation
	Class Weights	Balanced
	Max Iterations	1,000
	Solver	L-BFGS
	Feature Scaling	Standardization (z-score)
	Penalty Type	L1 (Lasso)
XGBoost (Extreme Gradient Boosting)	Learning Rate (η)	0.1
	Max Depth	6
	Number of Estimators	500
	Regularization Parameters (α, *λ*)	L1: 0.01, L2: 1.0
	Subsample Ratio	0.8
	Early Stopping Rounds	50
	Scale Positive Weight	1 (balanced classes)
	Tree Method	GPU Histogram

A classification task that uses the logistic function to predict the probability of a given instance belonging to a specific class uses the intercept and coefficients corresponding to feature values. Regularization techniques include L1 and L2 Regularization. Regularized Logistic Regression is a powerful tool for binary classification, particularly when dealing with large numbers of features or facing potential overfitting issues.

It improves the basic logistic regression model by adding a penalty term to the loss function, which helps manage issues related to overfitting and enhances the model's ability to generalize to unseen data. There are two primary types of regularization: L1 Regularization (Lasso) and L2 Regularization (Ridge). Key hyperparameters used here include Regularization Strength [\(\lambda \)], Class Weights, Max Iterations, Tolerance for Stopping Criteria, Solver, Feature Scaling, and Penalty Type. The benefits of regularized logistic Regression include handling multicollinearity, promoting sparsity (L1), improving generalization, and allowing model tailoring to specific datasets or problems. Challenges include hyperparameter tuning, which requires experimentation and can be computationally intensive. Regularized Logistic Regression is a robust and accurate extension of logistic Regression, particularly useful in complex, high-dimensional datasets, enhancing performance and reducing overfitting.

#### Extreme gradient boosting architecture

Extreme Gradient Boosting (XGBoost) (15, 16) is a powerful and efficient implementation of Gradient boosting that has gained popularity in machine learning competitions and practical applications due to its performance and speed. The algorithm consists of a boosting framework, where weak learners, typically decision trees, are combined sequentially to focus on hard-to-predict samples. It has two main components: the objective function, which measures the model's predictions aligning with actual values, and the regularization terms, which prevent overfitting and control the model's complexity. XGBoost uses gradient descent to optimize the objective function and has a built-in capability to handle missing values. Key hyperparameters used in this study are learning rate, maximum depth, sample proportion, sample_bytree, regularization parameters, number of estimators, scale positive weight, gap, early stopping rounds, and boosting type. These parameters help optimize performance and achieve model generalization, making it a preferred choice in many machine-learning scenarios. XGBoost is highly efficient and versatile for both classification and regression tasks, and it is adept at handling large datasets and feature-rich environments for impaction classification.

## Results

The data were analyzed using SPSS software ver 29.0, and mean and standard deviation were calculated. The Regression and ANOVA were also calculated. These results have been calculated for the segregated and grouped data sets. (mesial, vertical, horizontal, and distal types) A total of 303 OPG were analyzed. As briefly discussed, the analysis is linked to a regression model, which in turn enables us to come up with a predicted S/W ratio (with potential links to dental measurements), so to reiterate, the foundation of the compilation of predictors is thus far as follows: Distance from the apex to the lower border of the mandible, Space available distal to the 2nd molar, and the width of the 3rd molar, we will dissect the analysis into interpreting the statistical data for each category of impaction, and the inspecting each subcategory.

When we compare the S/W ratio, the lowest value (0.9267) is seen in the horizontal type, which shows that the space available diatal to the 2nd molar was the least in the horizontal type. This can be interpreted as the impaction type with the least potential for eruption in future years.

The regression equation obtained from the analysis is given as **{S/W ratio} = 1.082 - 0.0G4 ({Width of the 3rd molar}) + 0.08G({Space available distal to 2nd molar}),** so if we get values like the width of the impacted molar and the available space distal to the 2nd molar from the OPG, we can calculate the S/W ratio from the given impaction type. If the ratio is greater than 1.1, the chance of a lower 3rd molar eruption is good; if the ratio is less than 0.8, there is no chance of eruption existing5. The closer the R-value is to 1, the better the prediction of impaction from the formula will be. A very high R-squared value indicates that the model fits the data well.

The statistical method we employed in our study is regression analysis, which attempts to make significant findings concerning study correlations. Apart from regression analysis, the ANOVA model (Analysis of variance) is tested, which considers if the various groups' means are significantly different and is useful in establishing a predictive model (Table 2). The ANOVA Table 3 indicates that the prediction for the dependent variable (S/W ratio) using the suggested Regression model is statistically significant since the *p*-value for this model (*p* < 0.0005) is less than the significant level (*α* = 0.05), which implies that the regression model significantly predicts the outcome variable (S/W ratio).

**Table 2:** Comparison of all parameters for all impaction types with grouped data.

Types	Horizontal		Vertical		Mesial		Distal		All types grouped	
	Mean	Std. Dev	Mean	Std. Dev	Mean	Std. Dev	Mean	Std. Dev	Mean	Std. Dev
S/W ratio	.9267	.23090	1.1258	.22804	1.1291	.19097	1.0725	.25415	1.0373	.242
										67
Width of the 3rd molar	11.3695	.99307	10.6546	1.28717	10.2943	1.00468	10.5621	1.04203	10.8645	1.16
										654
Space available distal to 2nd molar	10.4145	2.29144	11.9053	2.47349	11.5586	1.94163	11.2138	2.53454	11.1292	2.36
										557
Distance from the apex to the lower border	18.4035	4.77804	12.4421	3.46166	14.3364	4.06064	11.7500	2.71980	15.3318	4.95
										901
Types	Horizontal		Vertical		Mesial		Distal		All types grouped	
The regression equation for the S/W ratio.	{S/W ratio} = 1.002–0.084({Width of the 3rd molar}) + 0.087({Space available distal to 2nd molar})		S/W ratio = 1.172–0.106({Width of the 3rd molar}) + 0.0G1(Space available distal to 2nd molar})		{S/W ratio} = 1.132–0.108(Width of the 3rd molar}) + 0.0G6({Space available distal to 2nd molar})		S/W ratio = 0.G43–0.084({Width of the 3rd molar}) + 0.0G0(Space available distal to 2nd molar})		{S/W ratio} = 1.082–0.0G4({Width of the 3rd molar}) + 0.08G({Space available distal to 2nd molar})	
Types	Horizontal		Vertical		Mesial		Distal		All types grouped	
R	.996		.990		.996		.996		.994	
R Square	.993		.980		.991		.992		.988	
Adjusted R Square	.993		.980		.991		.991		.987	
Std. Error of the Estimate	.01970		.03263		.01813		02457		.02714	

**Table 3:** “ANOVA table for all impactions regression model”.

Model		Sum of Squares	df	Mean Square	F	Sig.
1	Regression	17.564	3	5.855	7,948.385	.000
	Residual	.220	299	.001		
	Total	17.784	302			

Pearson correlation tests were presented in Table 4 to check the correlation between the dependent and the independent variables, and the results indicate that the S/W ratio has a strong significant positive correlation (*r* = 0.883 C *p* < 0.001) to the space available distal to the 2nd molar. While the S/W ratio has a significant moderate negative correlation (*r* = – 0.488 C *p* < 0.001) to the width of the 3rd molar, there is no correlation to the space available distal to the 2nd molar (*r* = −0.036, *p* = 0.266), which is not statistically significant.

**Table 4:** “Correlation analysis of parameters of all impactions”.

		S/W ratio	Width of the 3rd molar	Space available distal to 2nd molar	Distance from the apex to the lower border
Pearson Correlation	S/W ratio	1.000	−.488	.883	−.231
	Width of the 3rd molar	−.488	1.000	−.036	.308
	Space available distal to 2nd molar	.883	−.036	1.000	−.093
	Distance from the apex to the lower border	−.231	.308	−.093	1.000
Sig. (1-tailed)	S/W ratio	.	.000	.000	.000
	Width of the 3rd molar	.000	.	.266	.000
	Space available distal to 2nd molar	.000	.266	.	.053
	Distance from the apex to the lower border	.000	.000	.053	.

Moreover, the distance from the apex to the IAC has an uncertain significant correlation to the S/W ratio (*r* = −0.231, *p* < 0.001) and the width of the 3rd molar (*r* = 0.308, *p* < 0.001). Still, these correlations are weaker than those between the S/W ratio and the available space. It can be seen that there's strong proof against the idea of no correlations since the *p*-values for the correlation involving the S/W ratio are very low (*p* < 0.001). In contrast, the correlations between the width of the 3rd molar and the available space and distance from the apex have higher p-values, suggesting weaker proof of any correlations.

In summary, the S/W ratio significantly correlates with the space available distal to the 2nd molar and significantly negatively correlates with the width of the 3rd molar. The results recommend carefully applying these variables, which is important in dental procedures and treatment planning cases involving mandibular 3rd molar impactions.

### Mesial impaction group

R-Square (the coefficient of determination) signifies an overall measure of the strength of association. The Variance ratio in the dependent variable (S/w ratio) can be predicted from the independent variable was the Width of the 3rd molar C Space available distal to the 2nd molar. (R-Square = 99.1%) indicates that 99.1% of the variance in (S/w Ratio) scores can be predicted from the variable width of the 3rd molar C space available distal to the 2nd molar.

The standard error of the estimate was 0.01813, indicative of the average distance from which values are off the regression line. According to this model, we can deduce that the regression model is highly significant (*p* < 0.0001). This establishes that at least one of the predictors significantly aids in predicting the S/W ratio. The coefficient data provides an approximated coefficient for each predictor in the regression equation. The width of the third molar had a negative coefficient (−0.108), with a negative correlation implying that as the width increases in value, the S/W ratio decreases. The Space available distal to the 2nd molar has a positive coefficient (0.096); hence, a positive correlation indicates that more Space available leads to a higher S/W ratio (Table 1).

It is important to highlight in this study that the distance from the apex to the lower border of the mandible coefficient is negligible (0.000), numerically not of consequence (*p* = 0.429), and this parameter is not statistically significant.

### Horizontal impaction group

Again, in the regression model, the displayed R-squared value is 0.993%, which is quite high, illustrating that this model can explain 99.3% of the variance in the S/W ratio. A very high R-squared value indicates that the model fits the data well. The S/W ratio equation has been illustrated in the results above. The standard error of the estimate is 0.01970, indicating the average distance the observed values are off the regression line. This Table displays that the regression model is statistically significant (*P* < 0.001), indicating that, at the very least, one of the predictors contributes significantly to estimating the S/W ratio. As previously mentioned, the displayed coefficient table provides the approximated coefficients for each predictor in the regression equation. The width of the third molar has a negative coefficient (−0.084), showing us that as the width increases, the S/W ratio tends to decrease, expressing a negative correlation.

The Space available distal to the 2nd molar has a positive coefficient (0.087), showing that more Space available leads to a higher S/W ratio. The distance from the apex to the lower border also has a negative coefficient (−0.001), showing that the S/W ratio tends to decrease as the distance increases. However, this outcome seems to have marginal consequences compared to the other predictors. In the Horizontal impaction group, we can determine that the most significant predictors for the S/W ratio are the width of the 3rd molar and the Space available distal to the 2nd molar. At the same time, the distance from the apex to the lower border has a minimal outcome in predicting potential.

### Distal impactions

The R-squared value is 0.992, showing that this model explains approximately 99.2% of the variance in the S/W ratio. A very high R-squared value indicates that the model fits the data well. The regression equation for this model is expounded above in the results. The standard error of the estimate is 0.02457, indicating the average distance that the displayed values go off the regression line. The Table displays that the regression model is statistically significant with a *p*-value (*p* < 0.001), demonstrating that, at the very least, one of the predictors contributes to predicting the S/W ratio. The coefficient table gives approximated coefficients.

For each predictor in the regression equation. The width of the 3rd molar has a negative coefficient (−0.084). With a statistically significant negative correlation, showing that as the width increases, the S/W ratio tends to decrease. The Space available distal to the 2nd molar has a positive coefficient (0.090), with a positive statistically significant correlation. Therefore, more Space available leads to a higher S/W ratio.

The distance from the apex to the lower border of the mandible has a low positive coefficient (0.001), and it is not statistically significant since the *p*-value is very high (*p* = 0.681). Thus, this predictor does not significantly impact the S/W ratio in this particular model.

To summarize the Distal model, the most crucial predictors for the S/W ratio are the width of the 3rd molar and the Space available distal to the 2nd molar. The distance from the apex to the mandible's lower border does not affect the S/W ratio in this specific analysis.

### Vertical impactions

The R-squared value is 0.980, showing that this model accounts for about 98% of the variance in the S/W ratio. A very high R-squared value indicates that the model fits the data well. The standard error of the estimate is 0.03263, which shows the average distance from the observed values falls from the regression line. The data in the Table illustrates that the regression model is statistically significant with a *p*-value (*p* < 0.001), showing that at least one of the predictors significantly adds to estimating the S/W ratio. The coefficients table gives the approximated coefficients for each predictor in the regression equation. The width of the 3rd molar has a negative coefficient (−0.106), with a significant negative correlation indicating that as the width increases, the S/W ratio turns to decrease. The Space available distal to the 2nd molar has a positive coefficient (0.091), with a significant positive correlation illustrating that more Space available leads to a higher S/W ratio. The distance from the apex to the lower border of the mandible has a negligible coefficient (0.000), implying that this predictor does not have an important effect on the S/W ratio in the model.

## Machine learning results

The accuracy of regularised logistics regression is 78%.

HORIZONTAL class—A class prediction has a precision of 0.72, but an error rate of 28.1% is made, with the most frequently confused being mesial.

VERTICAL class: The prediction of a class has a precision of 0.57, but an error rate of 42.8% results in mispredictions, with the most common confusion being distal. The comparison shows that the horizontal type has a higher precision rate from our data (0.72), and the vertical type has a higher error rate (42.8%) compared to the horizontal (28.1%). Both classes are often confused with “mesial” and have a similar dark background with light grey text. These images appear to be part of a machine-learning model.

Precision in dental impaction predictions is critical, as it measures the ratio of true positive predictions to the total number of predicted positives. A precision score of 0.50 indicates that only 50% of the time when a model predicts a tooth class as impacted is correct, and 50% erroneously predicts impaction when not. This results in significant potential for misclassification, with 20% of cases mistakenly labeled as distal impactions, 20% misclassified as horizontal impactions, and 10% misclassified as vertical impactions. The low precision score suggests a need for improvements in the predictive model, including data quality, feature engineering, model complexity, and class imbalance.

## Extreme gradient boosting

ACCURACY OF THIS MODEL IS 75%, and The Horizontal class has a precision of 0.71 and a low mistaken prediction rate of 29%, with VERTICAL and MESIAL being the most common confusions. The VERTICAL class has a low precision of 0.36, with a high mistaken prediction rate of 63.6%, highlighting the challenge in accurately classifying instances. The MESIAL class has a precision of 0.43 but faces challenges due to confusion in the DISTAL, HORIZONTAL, and VERTICAL classes, which could be addressed to improve classification accuracy. The model's precision in identifying the DISTAL class is high at 0.71, while the VERTICAL class has a low precision at 0.36.

The graph showcases the performance of an eXtreme Gradient Boosted Trees Classifier, focusing on lift data for the “Class DISTAL,” showing an increasing trend between predicted and actual values, indicating the potential for improvement.

## Discussion

The study analyzed three parameters: the distance from the lower second molar to the mandible's anterior border, the mesiodistal width of the third molar, and the distance from the apex of the third molar root to the inferior border of the mandible. A regression analysis model was applied across four impaction types (18, 19). The study found that the mesiodistal width of the third molar and available space distal to the second molar are the primary predictors influencing the Width ratio in the vertical impaction group, overshadowing the significance of the distance from the apex of the root to the inferior border.

**Figure 5:**
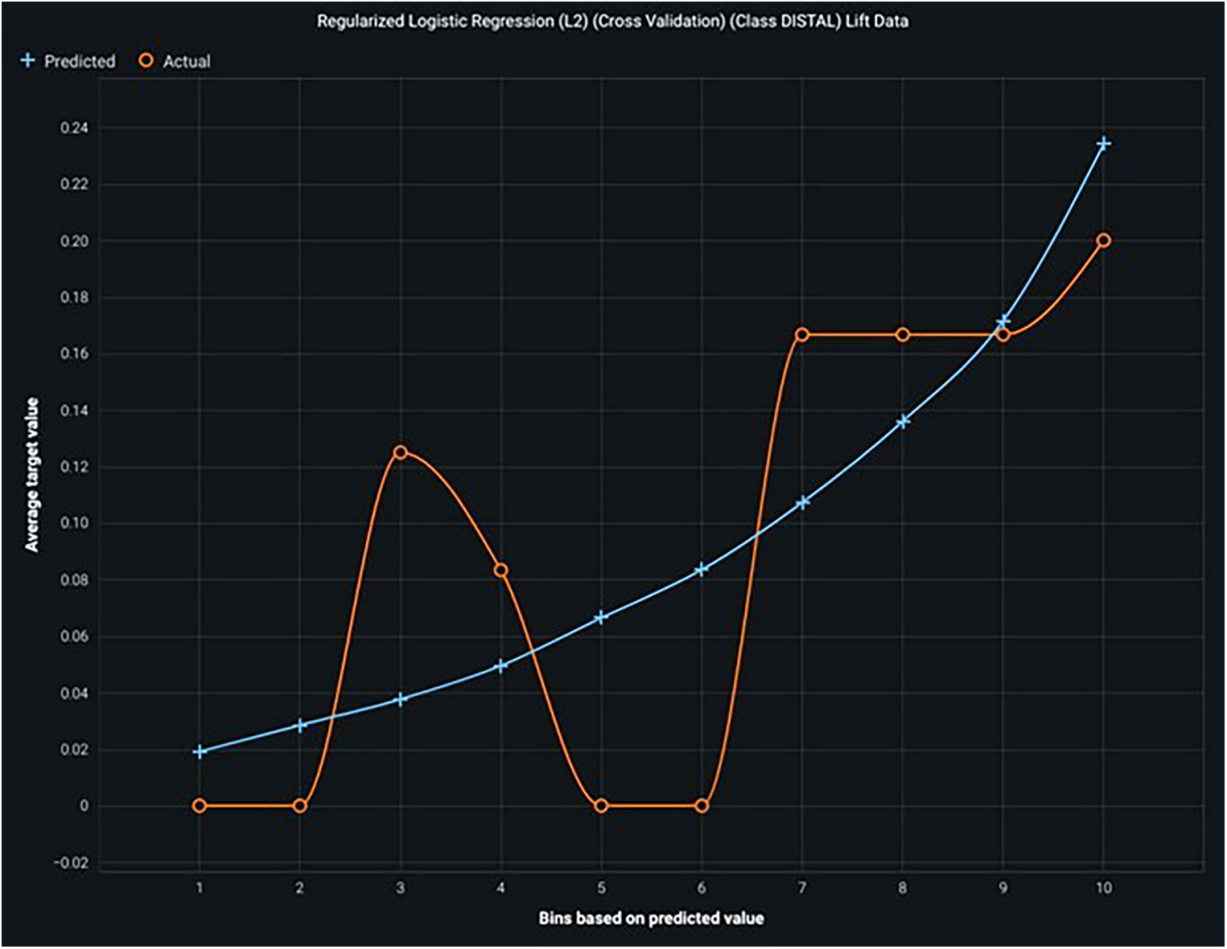
Shows the lift curve—with high lift, it shows good accuracy and prediction.

**Figure 6:**
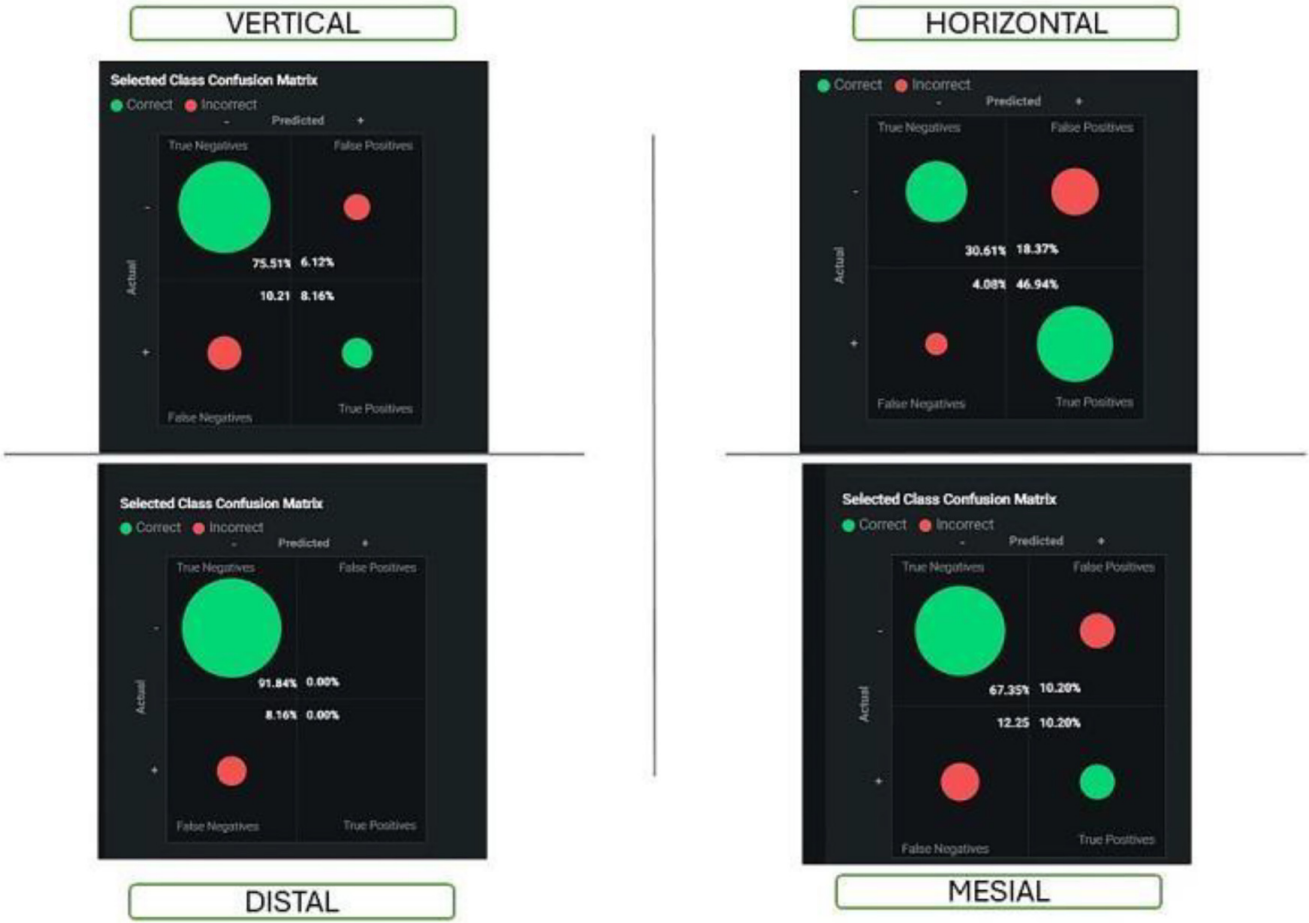
Shows the confusion matrix of all impaction groups in logistics regression.

**Figure 7:**
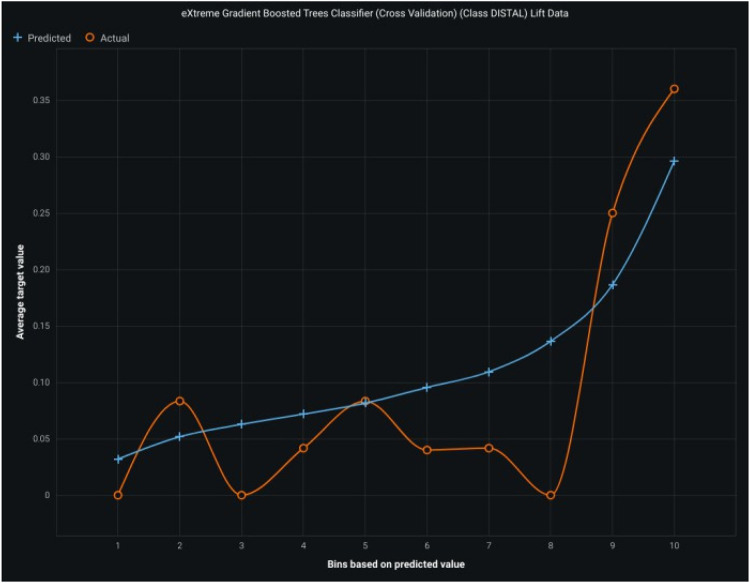
Shows the lift curve with high accuracy of the gradient boosting model.

**Figure 8:**
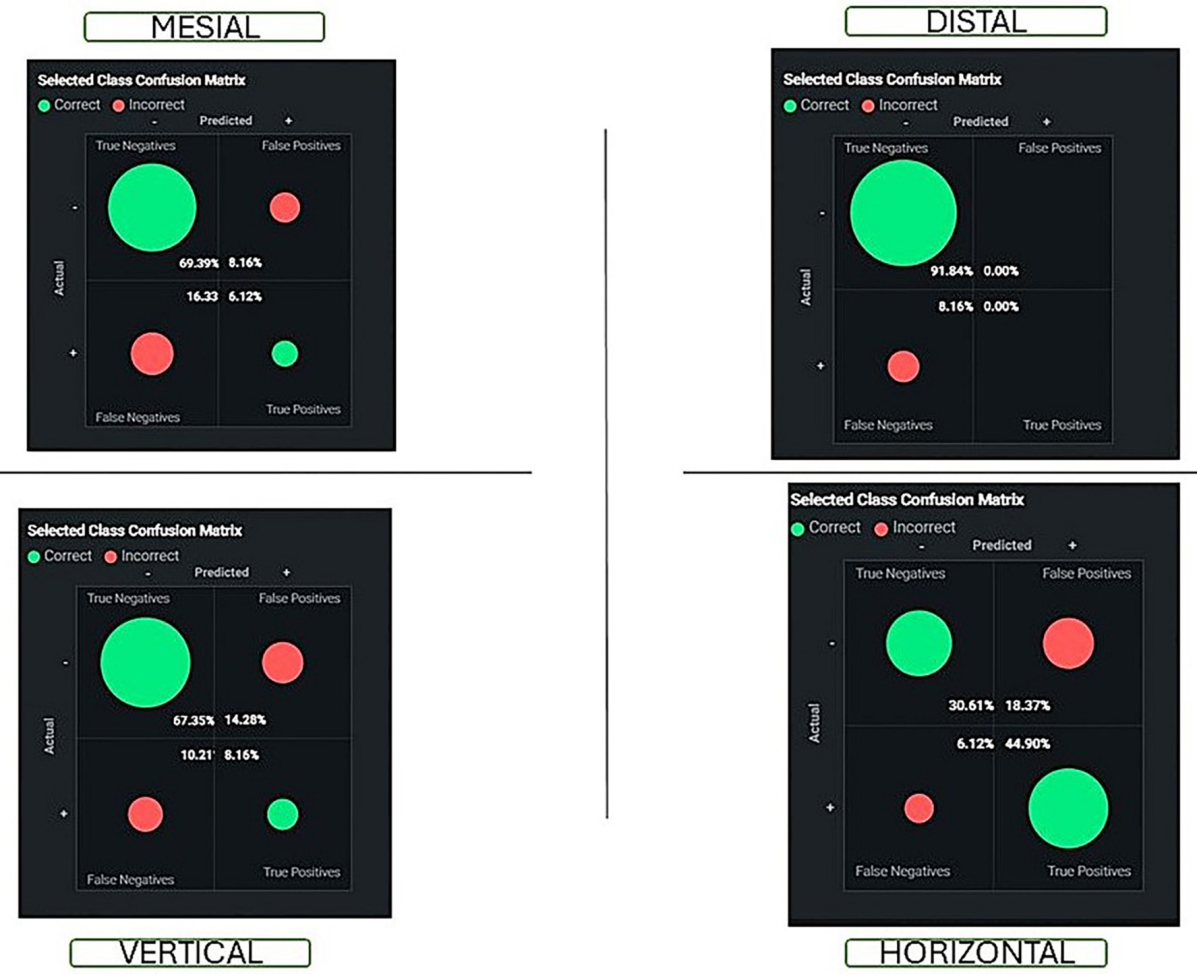
Shows the confusion matrix of all classes using the gradient boosting model.

When comparing our results to those of previous studies, we found that the Richardson study supported our results. In addition to that, Stefano Mummolo et al. (2023) (20) had results that were comparable to our results. Their control group (erupted third molars) had a space width ratio of 1.09, while the experimental group (impacted molars) had a space width ratio of 0.81. In another study by Al-Gunaid T.H. et al. 7 (2019) (19), it was found that in the control group (erupted), the space width ratio was 0.97. The space width ratio for the experimental group (impacted) was 0.75, which is still close to the results Richardson (1995) (21) and Mummolo6 et al. (2023)found that study evaluated extraction difficulty using the Pederson difficulty score and classified tooth images with a ResNet-34 model, achieving prediction accuracies of 78.91%, 82.03%, and 90.23%. Additionally, the YOLO-V4 model outperformed the Faster R-CNN in dental panoramic radiography, achieving 99.90% precision, 99.18% recall, and 99.54% F1 score, highlighting the advantages of deep learning methods for dentists similar to this study, Regularized logistic regression model achieved 75% accuracy in classifying dental impactions, with the HORIZONTAL class having a precision of 0.71 and a 29% error rate. The VERTICAL class had a low precision of 0.36 and a high error rate of 63.6%, often misclassified as DISTAL. The MESIAL class faced difficulties in differentiation, while the DISTAL class showed better performance with a precision of 0.71. The model's moderate accuracy suggests improvements in data quality, feature engineering, model complexity, and class balance (Figures 5–8).

The study suggests improving dental impact prediction models by enhancing the training dataset, enhancing feature engineering, experimenting with advanced modeling techniques, addressing class imbalance, continuously learning from new data, and collaborating with dental experts to understand the practical implications of model predictions. The training dataset for a model for dental impaction may not cover all clinical scenarios, limiting its performance (8, 22, 23). There are also confusions between classes, such as horizontal, vertical, and mesial, which could benefit from further clarification or unique features. The model's interpretability may be limited due to its complexity, which can hinder clinical adoption. Real-world variability, such as imaging conditions, classification criteria, and dynamic dental impaction, may not be captured in the dataset. The focus on precision may overlook the importance of recall, especially in clinical settings where false negatives can have severe implications (24, 25, 26).

Predicting tooth impactions can be effectively achieved by analyzing the S/W ratio, which is calculated using a regression equation from regression analysis. An S/W ratio below 1.1 indicates a high probability of impaction, while a ratio below 0.8 signifies a 100% likelihood of impaction. Long-term observation is advised for impacted mandibular third molars without pathology, with annual evaluations recommended for other impacted teeth. Surgical intervention is warranted when the tooth is obstructed by soft tissue or bone, with coronectomy—a method that retains the root while removing the crown—demonstrating fewer postoperative complications than complete extraction, with similar postoperative pain outcomes.

## Conclusions

Predicting and classifying dental impactions remains a challenging task. Although we gathered crucial information about predicting the eruption of third molars, we could improve and add to this critical area in dental research by layering upon this foundation and addressing other factors that influence eruption. All in all, the essence of our study is captured in its ability to aid dental practitioners in predicting the behavior of eruption of lower molars, thereby opening a window of opportunity in making more concise treatment plans that can lay out a plethora of options, understand the critical point of intervention and hopefully optimize treatments for patients by reducing the potential of complications.

This will be a useful reference for practitioners using the ratio in a practical setting. It will enable them to benefit from this research, extend the horizon of dental treatment possibilities, and provide a foundation upon which research and expertise can be dispersed.

The presented models demonstrate varying precision and error rates, highlighting the complexities arising from overlapping class characteristics. Enhancing data quality, refining feature selection, and using advanced modeling techniques are crucial for improving predictive capabilities in models. What emerged in our study was the most significant.

The parameters used to establish the space/width ratio were the distance from the second molar to the border of the anterior ramus and the mesiodistal width of the third molar. This highlights the need to record the parameters effectively in our clinical applications as practitioners and offers us valuable insight into how we might implement the treatment plan.

## Data Availability

The datasets presented in this article are not readily available because restrictions on hospital clinical data repository on ethical basis. Requests to access the datasets should be directed to Asok Mathew a.mathew@ajman.ac.ae.
